# Heat stress but not inbreeding affects offensive sperm competitiveness in *Callosobruchus maculatus*

**DOI:** 10.1002/ece3.667

**Published:** 2013-07-24

**Authors:** Emile Lieshout, Joseph L Tomkins, Leigh W Simmons

**Affiliations:** Centre for Evolutionary Biology, School of Animal Biology (M092), University of Western AustraliaCrawley, Australia

**Keywords:** Ejaculate size, heat shock, Hsp, inbreeding, paternity, sperm competition, stress

## Abstract

Environmental and genetic stress have well-known detrimental effects on ejaculate quality, but their concomitant effect on male fitness remains poorly understood. We used competitive fertilization assays to expose the effects of stress on offensive sperm competitive ability in the beetle *Callosobruchus maculatus*, a species where ejaculates make up more than 5% of male body mass. To examine the effects of environmental and genetic stress, males derived from outcrosses or sib matings were heat shocked at 50°C for 50 min during the pupal stage, while their siblings were maintained at a standard rearing temperature of 28°C. Heat-shocked males achieved only half the offensive paternity success of their siblings. While this population exhibited inbreeding depression in body size, sperm competitiveness was unaffected by inbreeding, nor did the effect of heat shock stress on sperm competitiveness depend on inbreeding status. In contrast, pupal emergence success was increased by 34% among heat-stressed individuals, regardless of their inbreeding status. Heat-shocked males' ejaculate size was 19% reduced, but they exhibited 25% increased mating duration in single mating trials. Our results highlight both the importance of stress in postcopulatory sexual selection, and the variability among stressors in affecting male fitness.

## Introduction

As the vehicle for male gametes, the ejaculate plays a central role in determining male reproductive fitness. The importance of both the sperm and nonsperm components (seminal fluid proteins) of the ejaculate in postcopulatory natural and sexual selection are now well recognized (Simmons and Fitzpatrick 2012). For example, sperm numbers, motility, and morphology all have effects on male fitness (Froman et al. 2002; Gage and Morrow 2003). As do the quantities of seminal fluid and accessory gland products that can have profound effects on sperm competition by modifying female reproductive behavior (Chapman 2001; Simmons 2001; Gillott 2003). Although many components of ejaculates have well-established genetic bases (Hales et al. 1989; Ducrocq and Humblot 1995; Swanson et al. 2001; Birkhead et al. 2005; Dowling et al. 2007; Dobler and Hosken 2009), estimates for heritability and additive genetic variance in sperm-competitive performance are generally modest (reviewed in: Simmons and Moore 2009). The influence of environmental effects on sperm competitive success is increasingly recognized (e.g., oviposition site availability: Eady et al. 2004; larval density, nutrition: Amitin and Pitnick 2007; adult density: Crean and Marshall 2008; sperm competition risk: DelBarco-Trillo 2011; immune insult: McNamara et al. 2013).

Through its well-established effects on ejaculate quality, stress is often presumed to be an important environmental source of variance in male fitness (Campbell et al. 1992; Pérez-Crespo et al. 2008; Hansen 2009). While the cause of stress can vary considerably (e.g., heat, excess reactive oxygen species, or physical handling), cellular responses to stress-related damage in affected tissues are remarkably consistent. Stress induces the expression of heat shock proteins (Hsp), molecular chaperones that repair cellular damage associated with stress (reviewed in Sørensen et al. 2003). Partly due to their resource requirements and demands on the transcriptional machinery, the expression of these gene products imposes its own costs (Feder et al. 1992; Krebs and Feder 1998), and the survival benefits of expression typically trade-off against other fitness components (Hoffmann 1995). While the adverse effects of heat stress on sperm production and function are well studied (Hansen 2009), knowledge about its consequences on other seminal components remains limited.

Hsp expression also appears to mitigate the consequences of genetic stress. A recent study in *Caenorhabditis elegans* showed that induced Hsp expression reduced the penetrance of a late-onset detrimental mutation (Casanueva et al. 2012). In a similar fashion, Hsp expression might be expected to counter the effects of the expression of detrimental alleles in homozygotes after inbreeding. Kristensen et al. (2002) found raised levels of Hsp70, a widely expressed and inducible chaperone, in unstressed inbred *Drosophila* larvae. While environmental stress usually worsens inbreeding depression (Armbruster and Reed 2005), inbred genotypes in *Drosophila buzzatii* show less of a decline in hatching success following heat shock than do outbred genotypes (Dahlgaard and Loeschcke 1997). However, attenuating effects of exposure to one stressor on susceptibility to another are not always supported. For example, in *Drosophila melanogaster*, the degree of inbreeding has no significant effect on heat stress survival (Dahlgaard et al. 1995). Although Hsp genes are highly conserved, their expression exhibits genetic variation in many species (Sørensen et al. 2003), and may thus show inbreeding depression. How interactions between intrinsic (genetic) and extrinsic stress affect trade-offs with other fitness components remains poorly understood.

In this study, we used the seed beetle *Callosobruchus maculatus* as a model to study how interactions between acute thermal and genetic stressors affect male fitness. Males transfer costly ejaculates that comprise 5–8% of their body mass (Savalli and Fox 1999). For females, these ejaculates are a source of water and accessory secretions that have complex effects on female fitness (Eady et al. 2007; Edvardsson 2007). As a pest of stored legumes, inbreeding is likely to occur “naturally” after colonization of new food sources. Inbreeding affects the number of sperm transferred per ejaculate (Fox et al. 2012). In densely infested legumes, metabolic processes may strongly elevate temperatures (Utida 1972), which larvae, developing within the beans, cannot escape. Although adult heat shock exposure is generally thought to induce temporary increases in Hsp expression, in zebrafish thermal stress during development produced a permanent increase in thermal tolerance at the expense of body size (Schaefer and Ryan 2006). Because males in *C. maculatus* typically attain high last-male sperm precedence (see Material and Methods), we examined the effects of thermal and genetic stress on this offensive sperm competitive performance. We manipulated stress by heat shocking one of two male siblings that were derived from inbred or outbred crosses, during the pupal stage. This developmental stage is the most resistant to heat stress (Johnson et al. 2010), and represents a period of major spermatogenesis and proliferation of secretory epithelia (Dumser 1980; Happ 1992). We also examined whether any reductions in sperm competitive ability result from changes in ejaculate size.

## Material and Methods

### Stock culture

Experimental animals were sourced from a large outbred population that originated from a stock culture held by the Stored Grain Research Laboratory of CSIRO (Canberra, Australia). Beetles were maintained at 28°C in an incubator (Binder KB 240, Germany) on black-eyed beans (*Vigna unguiculata*). In this population, a single generation of inbreeding causes genetic stress. This is shown by the observation that the offspring from brother–sister matings are significantly lighter at eclosion than outbred offspring (inbred males 2.6% lighter, *F*_1,1604_ = 9.8, *P* < 0.002 and inbred females 5.6% lighter, *F*_1,1777_ = 48.2, *P* < 0.001).

### Protocol

**Figure 1:**
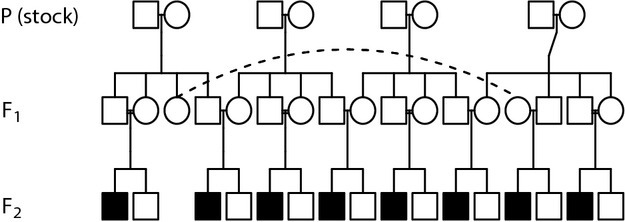
One of 15 replicate blocks within our experimental design. Stock virgins were single mated to produce four F1 families. Within each of these, two F1 siblings were mated to generate inbred F2 offspring, and two siblings were mated to beetles from other families to produce outcrossed F2 offspring. Shortly before emergence (day 26), F2 families were split: part of the pupae received a heat shock treatment (50 min on 50°C) while the remaining pupae were maintained at standard culturing conditions. Resulting F2 males were mated to females previously mated to irradiated males (not shown) to assess the effects of heat shock and inbreeding treatments on sperm-competitive ability. Dashed line indicates mating between nonadjacently depicted families. ○: females; □ males; ▪ heat-shocked males.

We used a split-family design to examine the effects of genetic stress (inbreeding) and environmental stress (heat shock during pupation) on sperm competitiveness of related males (Fig. 1). Parental virgins were derived from the population by haphazardly isolating infested beans in microtubes. Sixty pairs of virgins on the second day after emergence were formed and mated in microtubes. Females were then moved to 55 mL plastic vials (Techno Plas, Australia) with 40 black-eyed beans and allowed to oviposit until death. Infested beans were once again isolated in microtubes to assure virginity among F_1_ animals. Within each F_1_ family, a “brother–sister” cross was conducted and an outbred cross was set up using a donor female from the next family within the block (Fig. 1). In outbred populations, inbreeding coefficients increase most strongly in the first generation of inbreeding, which generally predicts the degree of inbreeding depression (Roff 1997). Animals used for these crosses were between 2 and 4 days old. Following mating in microtubes, females were allowed to oviposit until death on 40 black-eyed beans, which were then isolated to capture virgin F_2_ animals.

On the 26th day after the mating, clutches of inbred and outbred unemerged F_2_ individuals were randomly split between control and heat shock treatments. Preliminary trials indicated that this day precedes a 5-day period in which two thirds of emergences occur. This timing thus ensures that the majority of animals received heat shock treatment late in the pupal stage. Spermiogenesis in insects generally takes place during the final larval stages (i.e., pupal stage) (Dumser 1980), and spermatophore production in *C. maculatus* may be incomplete before 24 h postemergence (Eady and Brown 2000). Heat-shock events during this phase therefore are likely to affect sperm production and subsequent ejaculate competitiveness. We exposed pupae inside their beans, inside microtubes, to 50°C for 50 min in the aforementioned incubators. This treatment minimizes any water loss and is expected to induce strong cell-physiological responses, but exert minimal selection through mortality (Johnson et al. 2010). The effect of this stress was assessed by recording F_2_ eclosion success per randomly selected five beans for all experimental crosses within five blocks (20 families).

From each F_1_ family, four outbred non heat-shocked female F_2_ virgin offspring were collected for sperm competition trials. Females were always mated first to an irradiated virgin male (see below) and remated to an experimental male the next day. Female *C. maculatus* are generally polyandrous, but exhibit a refractory period after mating (Eady 1995). Females were given the opportunity to remate with the same male for 3 days in 15-min mating trials and were discarded if unsuccessful. Due to practical constraints, the ages at second mating of females, irradiated males, and experimental males were variable (median [iqr]: 5 [4–6]; 3.5 [3–5]; 5 [4–6] days, respectively), but controlled statistically. Following successful remating, females were placed in 55 mL plastic vials with 40 black-eyed beans and allowed to oviposit until death. For each female, the number of eggs and F_3_ emergences were counted.

### Irradiation

Irradiated males were produced by isolating infested beans from the stock population. Emerged males were kept in groups of 20 in ventilated vials (d × h = 24 × 64 mm) for 2 days before exposure to 60 Gy gamma radiation from a cobalt-60 source, over a period of 14 min and under nitrogen anesthesia (5 L min^−1^). Irradiated males' sperm remain functionally competent, but have DNA mutations that result in early embryonic mortality, so F_3_ could be attributed to experimental males (Parker 1970). Supplementary trials were conducted to confirm the efficacy of irradiation treatment on reducing fertilization ability. Fifty-eight 2-day-old females were successfully mated to either 2-day-old irradiated (27) or nonirradiated virgin males (31) and allowed to oviposit on 40 beans in 55 mL vials. Fecundity (egg number) and emergences were counted. Irradiation of males induced significant embryonic mortality in the offspring they sired (proportion emergence, median [iqr], control: 0.76 [0.61–0.82]; irradiated: 0.06 [0.03–0.11]; Wilcoxon rank sum test, *W* = 85.5, *P* < 0.001).

### Statistics

The consequences of stress on F_2_ eclosion success were examined by modeling the effects of experimental treatments (inbreeding, heat shock, and their interaction) with family as a random factor. To assess the effect of our treatments on the sperm competitiveness of F_2_ males, we analyzed F_3_ emergence data using generalized mixed-effects modeling with F_2_ fecundity as binomial totals. To address overdispersion, an observation-level random factor was included in the model. Graphical inspection of the data indicated between-family variation in the effect of heat shock treatment on F_3_ emergence. The fit of the full model was improved by including a random term that allowed the effects of heat shock treatment to vary by family (likelihood test, 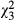 = 12.33, *P* = 0.006). The full model was weighted by fecundity and included fixed effects for heat shock treatment and inbreeding status, and five covariates to control for any effects of factors not fixed in the experimental design (female age at mating, duration of refractory period after the female's first mating, female body mass at emergence, and the ages of the first and second males at mating). Nonsignificant covariates were eliminated from the model by stepwise backward deletion. The full model also included interactions between heat shock treatment and inbreeding status, and between the ages of the first and second male at mating, but these were dropped due to nonsignificance. All analyses were conducted using R version 2.14.1 (R Development Core Team 2012), with the package “lme4” for mixed modeling (Bates et al. 2011).

## Results

**Figure 2:**
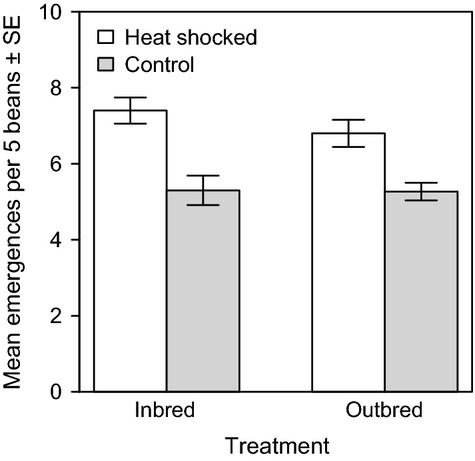
Effects of genetic (inbred vs. outcrossed) and environmental stress (heat shock vs. control) on F2 pupal eclosion success (number of emergences per five beans).

Heat-shock treatment induced significant viability benefits: stressed pupae showed a 34% increase in eclosion success compared to controls (7.1 vs. 5.3 emergences per five beans; type III ANOVA (analysis of variance) 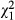 = 22.05, *P* < 0.001; Fig. 2); neither inbreeding nor its interaction with heat shock affected eclosion (both 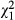 < 1.72, *P* > 0.19).

Forty-nine of 240 planned double mating trials were excluded due to failures to mate (*n* = 22), deaths before remating (*n* = 1), and other issues that prevented successful crosses. This reduction was not associated with any of the experimental treatments (χ^2^ test, 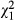 = 0.17, *P* = 0.68).

**Figure 3:**
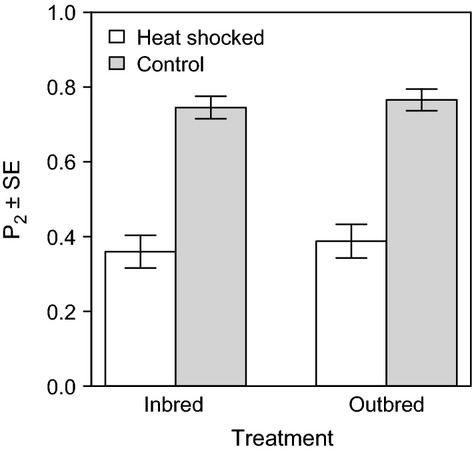
Effects of genetic (inbred vs. outcrossed) and environmental stress (heat shock vs. control) on the paternity over F_3_ offspring by experimental males (P_2_).

The reduced model revealed that only male heat shock treatment and female age at the first mating affected focal male paternity (P_2_) (Table 1). Heat-shocked males sired only half the proportion of offspring that non heat-shocked males gained (0.37 vs. 0.76, resp.; Fig. 3). Whether focal males were derived from in- or outbred crosses did not affect their sperm competitive ability (Fig. 3).

**Table 1:** Analysis of deviance table for a generalized linear mixed-effects model of the proportion paternity of the second male to mate (P_2_)

	β ± SE	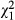	*P*
Male heat shock	−2.77 ± 0.38	54.22	0.000
Male inbreeding	−0.01 ± 0.28	0.00	0.974
Female age at first mating	0.20 ± 0.09	5.58	0.018

Random terms are not shown.

To examine the mechanism behind the decline in sperm competitive ability after heat shock, we determined ejaculate sizes of 23 heat-shocked and 23 control males between 1 and 3 days after eclosion. Prior to and after a timed mating with a 1-day-old virgin stock female, the male and female were weighed to the nearest 1 μg, and mass changes averaged. Heat-shocked males exhibited a 19% decrease in ejaculate mass (1.361 vs. 1.678 mg) despite 25% longer matings (592 vs. 474 sec) compared to control males (Table 2).

**Table 2:** Type II ANCOVA (analysis of covariance) results for the effects of heat shock treatment on transferred ejaculate mass and the duration of mating between virgin beetles

Term	Ejaculate mass			Mating duration		
	
	β	*F*_1,42_	*P*	β	*F*_1,42_	*P*
Heat shock	−495.34	11.40	0.002	135.51	8.30	0.006
Male body mass	0.06	12.15	0.001	−0.01	2.06	0.159
Female body mass	−0.01	2.74	0.105	−0.01	5.32	0.026

## Discussion

Thermal stress has well-known detrimental effects on male fertility. Our results indicate that the observed decline in offensive sperm competitiveness in stressed males was associated with reduced ejaculate size. Ejaculate size may affect sperm competitive success in several ways. The benefits of larger ejaculates in non heat-shocked males might simply derive from greater sperm numbers. Indeed, the pattern of sperm precedence in *C. maculatus* is consistent with sperm displacement from the spermatheca (Eady 1994). Sperm displacement might also be affected via seminal fluid components of the ejaculate, as in *Drosophila* (Wolfner 1997). However, the effect of quantitative changes in ejaculate components appears to be limited. Among virgin males, variation in ejaculate size has no effect on sperm precedence or female fecundity (Edvardsson and Tregenza 2005). Although mating causes significant ejaculate depletion, with roughly equivalent effects on ejaculate size and sperm numbers, the reduction in last-male sperm precedence due to depletion is far smaller than the reduction observed in this study (Eady 1995; Savalli and Fox 1999). Spermatozoa, spermatocytes, and spermatids do exhibit increased sensitivity to heat stress (Pérez-Crespo et al. 2008). We suggest therefore, that heat stress may have detrimental effects on gamete performance.

We further show that heat-stressed pupae exhibit a marked increase in eclosion success, indicative of up-regulated Hsp expression. Mild heat stress is often found to produce somatic benefits, including delayed senescence and increased longevity (Sørensen et al. 2003). Conversely, previous work indicates that reproductive effort reduces resistance to oxidative stress (Alonso-Alvarez et al. 2004), which is partly mediated by Hsps (Hartwig et al. 2009). The underperformance of control-treated individuals points to a common source of developmental mortality that is mitigated by increased Hsp expression. The pupal stage in holometabolous insects is accompanied by profound changes in transcriptional activity (Arbeitman et al. 2002). Mortality among larvae and unemerged adults in *C. maculatus* is substantial and heritable (Tran and Credland 1995). The pupal viability benefits associated with heat stress in this study are likely attributable to the roles Hsps play in stabilizing developmental processes (Takahashi et al. 2010) or counteracting the products of detrimental genes (Casanueva et al. 2012).

The evidence for increased investment in Hsps may provide a potential mechanism for the decrease in sperm competitive performance. While gametogenic and secretory tissues, and gametes may suffer direct stress-induced damage, sperm competitive performance may also be affected indirectly by a systemic or tissue-specific developmental trade-off between investment in reproduction and stress resistance. The costs of Hsp up-regulation on cellular functions are often more severe in reproductive cell types. In mice, Hsp expression is triggered at lower temperatures in spermatocytes than somatic reproductive cell types (Sarge 1995). Both germline and somatic reproductive tissues can further have Hsp expression profiles not found in nonreproductive tissues. In *Drosophila*, *Hsp23* and *Hsp27* are expressed in the secretory cells of the seminal vesicle and accessory glands, mainly during the pupal stage (Michaud et al. 1997). Expression of *Hsp23* is heat-stress inducible and associated with increased stress survival (Arrigo 1987). Up-regulation of similar Hsps might underlie the observed reduction in ejaculate size we observed in heat-shocked *C. maculatus*.

While body size is susceptible to genetic stress in our population, inbreeding had no direct effect on sperm competitive performance. Single generations of full-sib mating often reduce sperm competitive success (e.g., Simmons 2011), which is sometimes attributed to the relative complexity of spermatogenesis. Inbreeding depression in sperm competition traits has been found in other populations of *C. maculatus*, although these studies did not directly test offensive performance (Bilde et al. 2009; Fox et al. 2012). Fox et al. (2012) found that inbred males' ejaculates were similarly sized, but contain 17–33% fewer sperm. Given the relatively limited effects of severe sperm depletion on last-male sperm precedence (Eady 1995), any inbreeding depression in sperm numbers appears to have had no detectable effect on offensive sperm competitive success in our population.

Despite the absence of inbreeding depression in sperm competitive success, the effects of environmental stress on competitiveness could still depend on inbreeding status. First, Hsp genes, and the genes involved in their expression, may themselves be subject to inbreeding. Homozygosity at Hsp loci has been linked to reduced sperm numbers (Huang et al. 2002). Furthermore, synergistic effects of stressors are thought to result from competition between different sources of damaged proteins over *Hsp*s (Rutherford 2003). Inbreeding chronically raises Hsp expression (Kristensen et al. 2002). We hence expected the genome-wide stress of inbreeding to interact with the effects of heat shock, but found no such interactions. This result cannot be due to an overall lack of genetic variation in our population, because it exhibits inbreeding depression in other traits (this study; Tomkins et al. 2010). However, previous contradictory findings within populations of *C. maculatus* show that the effects of environmental stress on inbreeding depression in life-history traits strongly depend on the exact environmental conditions (Fox and Reed 2011; Fox et al. 2011). Despite the generality of the cellular heat shock response, different environmental stressors may exhibit varying interactions with genetic stress. Dahlgaard and Hoffmann (2000) found that inbreeding in *D. melanogaster* reduced resistance to several stressors, but not to heat knockdown. There are also indications that constitutively raised levels of Hsp due to inbreeding can reduce the costs of environmental stress (Dahlgaard and Loeschcke 1997), potentially masking the effects of other stressors such as heat shock.

In conclusion, we offer, to our knowledge, the first report of viability benefits concomitant with reductions in offensive sperm competitive performance induced by heat stress. This result appears consistent only with developmental up-regulation of Hsps. In *C. maculatus*, a key invertebrate model system for sexual selection and conflict, ejaculate components have important consequences for female fecundity, longevity, and remating propensity (Edvardsson 2007), highlighting the importance of environmental effects on variance in male postcopulatory success.
